# Chronic Syphilitic Salivation

**Published:** 1890-05

**Authors:** A. W. Furber

**Affiliations:** 80 Fortress Road, London, N.W.


### CHRONIC SYPHILITIC SALIVATION.

A. W. Furber, M. D., L. R. C. S. and L. D. S., says: I have for a long time had a—gentleman—patient under my care for disease of the teeth, and although my operations progressed favorably, I had many difficulties to contend with. The whole of my patient's teeth appeared to have a syphilitic taint, and with increased flow of saliva, amounting to chronic salivation. These were not the only troubles I had to surmount; but that which retarded my work most was the repeated recurrence of syphilitic ulcers of the sulcus and gums generally, which, though not painful to my patient, was still a source of considerable discomfort and militated greatly against the success of my operations. IODIA having come under my notice, I was inclined to give it a trial, and with the addition of a small proportion of liq. hydrarg. bi-chlor., taken daily before meals for a time—also used occasionally as a mouth wash—the salivation became normal, the mucous membrane assumed a more healthy state and the teeth generally looked like coming back to their original color. [80 Fortress Road, London, N.W.

